# Study of unripe and inferior banana flours pre-gelatinized by four different physical methods

**DOI:** 10.3389/fnut.2023.1201106

**Published:** 2023-06-19

**Authors:** Siwei Zhang, Kangyun Zhao, Fei Xu, Xiaoai Chen, Kexue Zhu, Yanjun Zhang, Guanghua Xia

**Affiliations:** ^1^College of Food Science and Engineering, Hainan University, Haikou, Hainan, China; ^2^Spice and Beverage Research Institute, Chinese Academy of Tropical Agricultural Sciences, Wanning, Hainan, China; ^3^Key Laboratory of Processing Suitability and Quality Control of the Special Tropical Crops of Hainan Province, Wanning, Hainan, China

**Keywords:** four different treatments, digestive properties, structure, starch, unripe and inferior banana flours

## Abstract

This study aimed to prepare the pre-gelatinized banana flours and compare the effects of four physical treatment methods (autoclaving, microwave, ultrasound, and heat-moisture) on the digestive and structural characteristics of unripe and inferior banana flours. After the four physical treatments, the resistant starch (RS) content values of unripe and inferior banana flours were decreased from 96.85% (RS2) to 28.99–48.37% (RS2 + RS3), while C∞ and k values were increased from 5.90% and 0.039 min^−1^ to 56.22–74.58% and 0.040–0.059 min^−1^, respectively. The gelatinization enthalpy (ΔHg) and I_1047/1022_ ratio (short-range ordered crystalline structures) were decreased from 15.19 J/g and 1.0139 to 12.01–13.72 J/g, 0.9275–0.9811, respectively. The relative crystallinity decreased from 36.25% to 21.69–26.30%, and the XRD patterns of ultrasound (UT) and heat-moisture (HMT) treatment flours maintained the C-type, but those samples pre-gelatinized by autoclave (AT) and microwave (MT) treatment were changed to C + V-type, and heat-moisture (HMT) treatment was changed to A-type. The surface of pre-gelatinized samples was rough, and MT and HMT showed large amorphous holes. The above changes in structure further confirmed the results of digestibility. According to the experimental results, UT was more suitable for processing unripe and inferior banana flours as UT had a higher RS content and thermal gelatinization temperatures, a lower degree and rate of hydrolysis, and a more crystalline structure. The study can provide a theoretical basis for developing and utilizing unripe and inferior banana flours.

## 1. Introduction

Unripe bananas are rich in starch, especially type-2 resistant starch (RS2). Bananas are typical respiratory leap fruits, and unripe bananas are susceptible to mechanical injury during ripening, resulting in varying degrees of inferior fruits during transportation and storage (1). About 15–20% inferior fruits from harvested bananas (2), which are difficult to sell as commercial products, resulting in resource waste. Unripe banana flours contain starch (78.19–81.82%), pectin (3.29–5.61%), protein (2.90–4.59%), lipid (0.32–0.57%), and ash (2.30–2.79%) (3). As bananas ripe, their starch is converted to free sugar, which increases postprandial blood glucose levels upon consumption (4). Inferior and unripe bananas were the fruits that were immature and had suffered mechanical damage, which can be used for prepare flours. For example, Fu et al. (5) found that the unripe banana flours supplementation could lower glycaemic index (GI) and insulin levels.

Some physical treatments have a great potential in food modification, considering their safety, environmental friendliness, and cost effectiveness, as well as the absence of chemical waste generation and enhancing product quality, compared to traditional thermal processing methods. Physical treatments, such as autoclaving (AT), microwave (MT), and ultrasound (UT) treatment, have been applied for starch modification. Raungrusmee et al. (6). assessed effects of ATs on resistant starch (RS) production from rice starch. Kumar et al. (7) focused on the effect of MT on the physicochemical, morphological, structural and rheological properties of starch. Kaur et al. (8) found an increase in rapidly digestible starch (RDS) and RS level after UT. Wang et al. (9) revealed the mechanism by which UT influences the structural properties of starch. A few studies have focused on the physical treatment of banana flours and starch. Liao et al. (10) used an AT to modification banana flours and found that the digestibility of the pre-gelatinized banana flours was higher than that of the native flours. Bi et al. (3) determined the *in vitro* digestive properties of cooked banana flours. Although many studies have been reported on the physical treatment of banana starch, only some studies have investigated the effects of more than three physical treatment methods on banana flours and compared its structure and digestive characteristics.

Therefore, this study aimed to prepare the pre-gelatinized sample and compare the effects of AT, MT, UT, and heat-moisture treatment (HMT) on unripe and inferior banana flours. Using *in vitro* digestion experiments, we analysed the effects of different methods on digestion with respect to enzymolysis. The thermal stability and structure of starch before and after the different treatments were characterized using differential scanning calorimetry (DSC), Fourier transform infrared spectroscopy, X-ray diffraction (XRD), and scanning electron microscopy (SEM). In this study, the interactions of different components of unripe and inferior banana flours were investigated, providing a solid foundation for the effective utilization of unripe bananas and a theoretical basis for the effects of different treatments on starch digestibility.

## 2. Materials and methods

### 2.1. Materials

The unripe and inferior banana fruits chosen as material were the *Musa sapientum Linn. ABB Dajiao* variety collected in July 2021 and purchased from the Xinglong Central Market (Hainan, China). The unripe and inferior bananas were the fruits that were immature and had suffered mechanical damage.

### 2.2. Unripe and inferior banana flours preparation (NC)

Unripe and inferior bananas were peeled and chopped into pieces. After drying in a 40°C drying oven and shattering, the samples were passed through a 60-mesh screen to obtain unripe and inferior banana flours, then stored in a desiccator, and labeled NC.

### 2.3. Treatment of unripe and inferior banana flours with different technologies

#### 2.3.1. Unripe and inferior banana flours by AT

AT was preformed based on the methodology described in a previous study (11): unripe and inferior banana flours was added to deionized water until the moisture content reached 30%, and 30 g of the moistened unripe and inferior banana flour samples were placed in a 250 mL conical flask. The conical flask was sealed, placed in a pressure vessel (G154DW, Xiamen Zhiwei Instrument Co., China), and heated at 120°C (0.12 MPa) for 15 min. After cooling until the sample attained 25°C, the sample were stored at 4°C for 24 h to enhance the formation of retrograded resistant starch. The samples were then placed in a 40°C drying oven until constant weight. The resulting product was milled into powder, sieved through a 60-mesh screen, and labeled AT.

#### 2.3.2. Unripe and inferior banana flours by MT

MT was performed according to the method established in a previous study (12), with some modifications: unripe and inferior banana flours was added to deionized water until the moisture content reached 30%, and 30 g of the moistened unripe and inferior banana flours sample was placed in a petri dish, sealed, and placed in a microwave oven (Guangdong Galanz Group Co., China) for 80 s at a power level of 5 W/g. After cooling until the sample attained 25°C, the sample was stored at 4°C for 24 h and then dried in a 40°C drying oven until constant weight. The resulting product was milled into powder, sieved through a 60-mesh screen, and labeled MT.

#### 2.3.3. Unripe and inferior banana flours by UT

30 g of the moistened unripe and inferior banana flours were suspended in distilled water (90 mL) using a modified version of the methodology described in a previous study (13). The slurry was treated using an ultrasonic probe processor (JY92-IIDN, Ningbo Xinzhi Biotechnology Co., China) equipped with a 12-mm ultrasonic horn. Ultrasound treatment was performed in a pulsed mode (2 s on and 2 s off) for 15 min with a nominal power of 300 W. After cooling until the sample attained 25°C, the sample was stored at 4°C for 24 h. Subsequently, the pre-gelatinized sample was dried in a 40°C drying oven until constant weight, milled into powder, sieved through a 60-mesh screen, and labeled UT.

#### 2.3.4. Unripe and inferior banana flours by HMT

The moistened unripe and inferior banana flours (30 g) were suspended in distilled water (90 mL), placed in a boiling water bath for 20 min, and stirred until a paste was formed. After cooling until the sample attained 25°C, the sample was stored at 4°C for 24 h. Subsequently, the pre-gelatinized samples were dried in a 40°C drying oven until constant weight, and the resulting product was milled into powder, sieved through a 60-mesh screen, and labeled HMT.

### 2.4. Analysis of composition and apparent amylose content

The starch, protein, lipid, ash, and pectin content of samples were determined according to the AOAC Official Methods of Analysis methods (14). The soluble sugar content was determined using anthrone colorimetry (15). The pectin content was determined by the spectrophotometry method as Bi et al. reported (3). The analysis of apparent amylose content was based on measuring absorbance at 620 nm using an ultraviolet–visible spectrophotometer (16).

### 2.5. *In vitro* digestibility of samples

As stated in the study by Englyst et al. (17), the proportions of slowly digested starch (SDS), RDS, and RS were calculated with slight modifications. A mixture of enzyme solution including 20 U/mL of porcine pancreatic α-amylase and 225 U/mL of amyloglucosidase were added to 20 mL of sodium acetate buffer (0.1 M, pH 5.2) along with 200 mg of sample. In a 37°C water bath, the combined solutions were incubated. At 20-and 120-min intervals, 70% ethanol (20 mL) was added to the supernatant (0.5 mL) to inactivate the enzymes. The supernatant from this solution was collected, and the glucose content was determined using the glucose oxidase-peroxidase method and a spectrophotometer (UV-2700, Shimadzu Co., Japan) at 510 nm. The RDS, SDS, and RS contents of samples were estimated as follows:


$$RDS=\frac{\left(G_{20}-G_{0}\right)\times 0.9}{TS}$$

$$SDS=\frac{\left(G_{120}-G_{20}\right)\times 0.9}{TS}$$

$$RS=\frac{TS-RDS-SDS}{TS}$$

where G_20_ (%) is the glucose content released within 20 min, G_120_ (%) is the glucose content released after 120 min, G_0_ (%) is the free glucose content, and TS (%) is the total starch content.

### 2.6. Kinetics characterization of *in vitro* starch digestibility

The *in vitro* digestibility kinetics of starch were determined using the method reported by Goñi et al. (18) with slight changes. Sodium acetate buffer solution (pH = 5.2, 15 mL) was mixed with 200 mg samples. Then, 15 U/mL of glucosidase, 290 U/mL of α-amylase from porcine pancreatic (total 10 mL), and seven glass were added to the solution. The mixed liquids were allowed to react on a stirred bed at 37°C. Absolute ethanol (4 mL) was added to the supernatant (0.5 mL) at 10, 20, 30, 60, 90, 120, and 180 min. This solution was centrifuged at 6,000 × g for 15 min at 3°C, and the supernatant was transferred to glucose oxidase–peroxidase mixture to analyse the glucose content. The equilibrium concentration (C∞) (%) and speed rate constant (k) (min^−1^) were obtained from the enzyme hydrolysis curves, and the first-order formulas were as follows (19):


$$C=C_{\infty }\left(1-e^{-kt}\right),\;C_{\infty }\leq 100\%$$

$$AUC=C_{\infty }\left(t_{f}-t_{0}\right)-\left(\frac{C_{\infty }}{k}\right)\left[1-\exp-k\left(t_{f}-t_{0}\right)\right]$$

where AUC is the area under the fitted curve, t_0_ and t_f_ are the initial and final hydrolysis times, respectively, and t is the time for the *in vitro* digestibility kinetics (min).

### 2.7. Predictive glycaemic index of starch

The area of the *in vitro* digestibility kinetics curve was calculated as the hydrolysis index (HI), and the estimated GI corresponding to white bread as a reference was calculated using the following equation (19):


$$HI=\frac{AUC\left(sample\right)}{AUC\left(whitebread\right)}$$

GI=39.71+(0.549HI)

### 2.8. Thermal properties

The thermal characteristics were determined using the methods described in a previous study (20). The samples were prepared by premixing the flours with distilled water (w/w, 1:3) in a sealed glass vial, stored at 20°C for 24 h before analysis. An approximately 3 mg (dry basis) sample, scanned in the 40–120°C at a slow heating rate of 10°C/min, was measured using a differential scanning calorimeter (Q2000, TA Co., United States). The onset (To), peak (Tp), and conclusion temperatures (Tc) and gelatinization enthalpies (ΔHg) of starch gelatinisation were calculated. The enthalpy was calculated based on the weight of the dry-based starch.

### 2.9. Fourier transform infrared spectrum

Fourier transform near-infrared spectrometer (Nicolet iS20, Thermo Fisher Co., United States) was used to obtained the short-range order structure (16). A deconvoluted straight line was used to baseline-correct the data at 1200 cm^−1^ and 800 cm^−1^. A 26 cm^−1^ half-band width and a 2.4 enhancement factor with triangular apodization were used. The absorbance ratio at 1047/1022 cm^−1^, 995/1022 cm^−1^ was calculated.

### 2.10. X-ray diffraction

The method of analysis was based on Zhang et al. (20). The samples were investigated with the X-ray diffractometer (Smartlab, Rigaku Corporation, Japan) equipped with a copper tube, which operated at 40 kV and 200 mA. Diffraction patterns were recorded at 15°/min, step length = 0.02°, scanning range = 4–40° (2°), scattering slit width = 1°, and slit width = 0.02 mm. Relative crystallinity (RC) was calculated using fitting software, which is the ratio of the area beneath the crystalline peak to the entire area.

### 2.11. Scanning electron microscopy

The prepared samples were analysed as described in a previous study (21). The samples were spread on metal stubs with adhesive carbon tape and coated with a gold layer using a sputtering coater. SEM analysis was performed using a desk-type microscope (Phenom ProX, Phenom-World Co., Netherlands) at an accelerating voltage of 15 kV.

### 2.12. Statistical analysis

One-way analysis of variance (ANOVA) was used to test the statistical significance of the differences in the indicators. ANOVA was followed by Duncan’s multiple difference test, and *p* < 0.05 was considered statistically significant. The curves were plotted using Origin 2021 software, and all data were tested in triplicates.

## 3. Results and discussion

### 3.1. Analysis of composition and amylose content

The chemical composition of the samples was listed in Table 1. The starch content of NC was 90.40%, and it contained 0.23% soluble sugar, 2.34% protein, 0.43% lipids, 1.76% ash, 3.15% pectin, and 30.3% amylose. However, the starch contents of AT, MT, UT, and HMT ranged between 75.18–82.56%. AT, MT, UT, and HMT contained 8.49–15.45% of soluble sugar, 2.13–2.35% of protein, 0.23–0.33% of lipids, 1.64–1.76% of ash, 2.85–3.02% of pectin, and 31.9–33.8% of amylose content. There was no significant difference in the ash content between NC and AT, MT, UT, and HMT. The starch content of the pre-gelatinized samples was significantly lower than that of the NC, whereas the soluble sugar and amylose contents were significantly increased, and the amylose contents differed significantly among the treatment methods (*p* < 0.05). MT had the highest amylose content, followed by UT, whereas the lowest was found in HMT. The trend of increased amylose content might because the glycosidic bonds of the starch chains were severely affected during treatment, resulting in the break of glycosidic bonds and an increase in soluble sugar and amylose contents, while the increase trend of amylose was opposite to that of soluble sugar.

**Table 1:** Chemical composition of the five samples.

Samples	NC	AT	MT	UT	HMT
Starch (%)	90.40 ± 0.23a	81.78 ± 0.07c	82.21 ± 0.21b	82.56 ± 0.35b	75.18 ± 0.35d
Soluble sugar (%)	0.23 ± 0.03d	9.75 ± 0.15b	8.49 ± 0.35c	8.79 ± 0.19c	15.45 ± 0.26a
Protein (%)	2.34 ± 0.09ab	2.47 ± 0.14a	2.13 ± 0.12b	2.35 ± 0.18ab	2.17 ± 0.19ab
Lipid (%)	0.43 ± 0.03a	0.40 ± 0.01a	0.42 ± 0.08a	0.28 ± 0.08b	0.33 ± 0.02ab
Ash (%)	1.76 ± 0.03a	1.76 ± 0.07a	1.64 ± 0.08a	1.71 ± 0.04a	1.65 ± 0.00a
Pectin (%)	3.15 ± 0.07a	2.94 ± 0.05bc	2.85 ± 0.05 cd	3.02 ± 0.05b	2.76 ± 0.06d
Amylose content (%)	30.3 ± 0.18d	32.5 ± 0.37b	33.8 ± 0.07a	33.5 ± 0.17a	31.9 ± 0.15c

### 3.2. *In vitro* nutritionally relevant starch fractions

The RDS, SDS, and RS contents of the samples were listed in Table 2. The RDS, SDS, and RS of NC were 1.42, 1.73, and 96.85%, respectively. The high RS content in NC demonstrates that unripe and inferior banana flours are a potential source of RS. The RS content of unripe and inferior banana flours in this study were higher than that reported in a previous study (47.25%) (22), these differences may be ascribed to starch granules size, amylose/amylopectin ratio, crystalline properties, starch source, or cooking condition.

**Table 2:** *In vitro* digestibility and composition of the five samples.

Samples	NC	AT	MT	UT	HMT
RDS (%)	1.42 ± 0.10e	40.56 ± 0.13b	37.57 ± 0.28c	26.07 ± 0.53d	44.22 ± 0.39a
SDS (%)	1.73 ± 0.02e	21.69 ± 0.41c	19.82 ± 0.18d	25.56 ± 0.33b	26.79 ± 0.15a
RS (%)	96.85 ± 0.89a	37.75 ± 0.24d	42.61 ± 0.14c	48.37 ± 0.34b	28.99 ± 0.45e
C_∞_ (%)	5.90 ± 0.90e	68.68 ± 1.42b	62.39 ± 2.21c	56.22 ± 2.23d	74.58 ± 1.63a
k (min^−1^)	0.039 ± 0.007b	0.051 ± 0.004a	0.049 ± 0.006ab	0.040 ± 0.004b	0.059 ± 0.003a
HI	6.26 ± 0.49e	78.95 ± 0.51b	70.20 ± 1.09c	60.35 ± 0.81d	85.25 ± 0.47a
GI	43.15 ± 0.27e	83.05 ± 0.29b	78.25 ± 0.60c	72.30 ± 0.45d	86.51 ± 0.26a

The RDS, SDS, and RS content differed significantly among the different treatment methods (*p* < 0.05). The RDS values of the different pre-gelatinized samples ranged from 26.07–44.22%. The HMT sample showed the highest SDS contents (26.79%), followed by the UT, AT, and MT samples (25.56, 21.69, and 19.82%, respectively). Noteworthily, SDS is digested completely in the small intestine, providing a sustained and prolonged glucose release, and has a moderate postprandial glycemic response compared to RDS (23). Hence, HMT sample is a food material with potential health benefits. UT exhibited the highest RS content (48.37%), followed by MT (42.61%) and AT (37.75%), whereas the lowest RS content was observed for HMT (28.99%) (Table 2). These differences were attributed to the effects of the four treatments. According to a previous study (13), starch chains could be broken by strong shear force and high temperature during UT, which aided UT increasing retrograded starch (RS3) values and improving digestion resistance. Another study stated that starch granules were instantly heated and absorbed energy in much less time during MT (24), which might cause the breakdown and change in the structure of amylopectin chains, leading to lower RS content than that in UT. Deka et al. (12) stated that autoclaving causes the breakdown of hydrogen bonds, causing the displacement of adjacent double-helices and loss of crystallinity. In the HMT, starch was promoted by disrupting the crystalline and helical structures, followed by the re-associating the disrupted crystals and mobility of amorphous regions, resulting in inadequate RS content in HMT (25). Similar results for RS content have also been reported, ranging from 32.14–31.83% (26), 9.22–6.75% (27), and 31.90–26.30% (28) after AT, MT, and HMT, respectively.

Following the various treatments, the RDS and SDS contents of pre-gelatinized samples increased compared to those in the NC, whereas the RS contents significantly decreased (*p* < 0.05). Compared with the NC, AT, MT, UT, and HMT showed a reduction in RS content by 59.10, 54.24, 48.48, and 67.86%, respectively. These results indicated that most RS2 in NC was converted into RDS and SDS in AT, MT, UT, and HMT after treatment. Although the RS content of the pre-gelatinized samples was significantly reduced, RS form changed from type-2 to type-3. A previous study (29) reported that normal cooking tends to destroy the structure of starch particles for most sources of RS2, potentially leading to gelatinization, thus increasing their digestibility. According to studies by Haralampu et al. (30) and Zhen et al. (31), RS3 is formed by disruption of the granule structure with heating, and slow recrystallization of starch molecules upon cooling or dehydration. RS3 shows considerable thermal stability, withstands heat treatment during cooking, and is widely used as food material. In this study, 28.99–48.37% of the RS remained after the four treatment methods, which can be used as a heat-stable component to maintain nutritional functioning during the preparation of food products.

### 3.3. *In vitro* digestive kinetics of treatment samples

The C∞ value represents the potential digestibility (%), and *k* is the digestion rate (32). The C∞ value for NC was 5.90%, and *k* was 0.039 min^−1^, consistent with the results reported in a previous study, which were 5.07% and 0.069 min^−1^ (33), respectively.

The C∞ and *k* of the various treatment samples differed significantly (*p* < 0.05), ranging from 56.22–74.58% and 0.040–0.059 min^−1^. HMT had the highest C∞ and *k*, followed by AT and MT, whereas the lowest was found for UT (Table 2). The results indicated that the degree and rate of hydrolysis of HMT samples were higher than those of the other samples. According to Sudheesh et al. (34), this finding could be explained by the higher digestibility of the pre-gelatinized samples, which can be attributed to the lower relative crystallinity and weak crystalline structure, implying that HMT methods may result in an increased loss of particle crystal structure.

The C∞ and *k* of the different treatment samples were higher than those of NC. As Li et al. (35) and Aaliya et al. (36) reported in previous studies, the significant differences in the C∞ and *k* due to the partial disruption of organized chain structures by different treatment and the starch crystallization properties, which could promote the attack of the enzymes into the interiors of starch granules, resulting in C∞ and *k* of the various treatment samples being significantly higher than those of NC. Furthermore, this may be due to the non-catalytic combination of protein and amylase, i.e., the contact between amylase and starch is reduced, leading to a decrease in digestibility (37).

### 3.4. Glycaemic index analysis

As shown in Table 2, the HI and GI values for NC were 6.26 and 43.15, respectively, which was a low GI food, as previously reported (38). The order of HI and GI of the pre-gelatinized samples were as HMT (85.25 and 86.51, respectively) > AT (78.95 and 83.05, respectively) > MT (70.20 and 78.25, respectively) > UT (60.35 and 72.30, respectively). The results indicate that MT, UT, AT, and HMT are high-GI foods (GI > 70). The HI and GI values of the different treatment groups were remarkably higher than those of NC (*p* < 0.05). According to Xie et al. study (39), the difference in digestibility due to the different molecular orders of the samples caused by the different treatment methods. The rise in soluble sugar content is indicative of an increase in GI. Another study (40) reported a similar result for the amaranth starch in which the GI value (91.2) of heat treatment starches was significantly higher than that of a native sample (87.2).

### 3.5. Thermal properties

**Figure 1:**
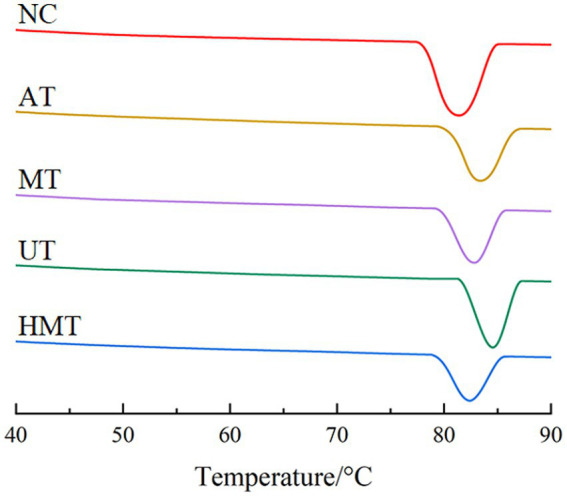
Thermograms of five samples.

The To, Tp, Tc, and ΔHg of NC were 78.17°C, 81.67°C, 85.09°C, and 15.19 J/g, respectively (Table 3 and Figure 1). Similar gelatinization temperatures and enthalpies have been reported in banana flours with range 64.67–71.21°C and 0.068–10.12 J/g (41), respectively.

**Table 3:** Changes in thermal properties of the five samples.

Samples	To (°C)	Tp (°C)	Tc (°C)	ΔHg (J/g)
NC	78.17 ± 0.37e	81.67 ± 0.25c	85.09 ± 0.16d	15.19 ± 0.07a
AT	80.14 ± 0.13b	83.74 ± 0.23c	86.97 ± 0.25b	12.75 ± 0.13c
MT	79.60 ± 0.22c	83.00 ± 0.27b	85.55 ± 0.31c	12.84 ± 0.22c
UT	81.51 ± 0.13a	84.45 ± 0.26a	87.81 ± 0.21a	13.72 ± 0.17b
HMT	79.04 ± 0.26d	82.34 ± 0.11b	85.34 ± 0.10 cd	12.01 ± 0.10d

The To, Tp, Tc, and ΔHg of pre-gelatinized samples ranged from 79.04–81.51°C, 82.34–84.45°C, 85.34–87.81°C, and 12.01–13.72 J/g, respectively. The gelatinization temperatures of the pre-gelatinized samples were of the order UT > MT > AT > HMT. According to Zhang et al. study (42), UT had more perfect crystals and crystal structure than those in MT, AT, and HMT. Based on Wang et al. (9), ultrasound produces a cavitation effect that loosens the internal structure of starch and produces an increased number of broken molecules. Compared with MT, AT and HMT, UT broken molecules, such as amylose, form crystalline nuclei, which had more crushing degree of starch, resulting more crystal nucleus, and the recombination and recrystallization of starch was faster during the short order retrogradation stage, and further retrogradation on the basis of the existence of crystallization nucleus during the long order retrogradation. Therefore, the RS content in UT was the highest among the four physical methods, and the RS content by AT, UT, and HMT was consistent with the intensity trend (MT > AT > HMT). The crystalline nucleus induces starch chains, proteins, and pectin to form an increased number of perfect crystal structures in UT, which requires high temperatures to unwind the double-helices and high energy to disrupt a certain number of ordered structures in the crystalline region.

The To, Tp, and Tc values of the different treatment samples were notably higher than those of NC, which could be explained by the rearrangement to form a perfect crystalline structure in the treatment samples. According to Zhang et al. (43) study, the gelatinization temperatures increased from 79.2–88.9°C to 84.0–93.6°C after extracted treatment of jackfruit, which was consistent with pre-gelatinized sample results. The ΔHg significantly decreased from 15.19 J/g to 12.01–13.72 J/g. As reported by another study (44), higher ΔHg value could be attributed to the larger proportion of double-helices in the granules’ crystalline domains, indicating the existence of more double-helices in the crystalline domains of NC than those of AT, MT, UT, and HMT samples; this proved that the different treatment methods impact the crystal region. Starch synthesis in plants is mediated by enzyme classes that form an increased number of double-helices in the NC (45). The double-helix of the pre-gelatinized samples was affected by various treatments: the cavitation effect of UT causes the double-helix structures to unwind; AT produce high pressure and heat destroy the structure; MT shake the water molecules and generate heat; and HMT generate heat and shear effect, thus decreasing the number of double-helix structures.

### 3.6. Fourier-transform infrared spectroscopy

**Figure 2:**
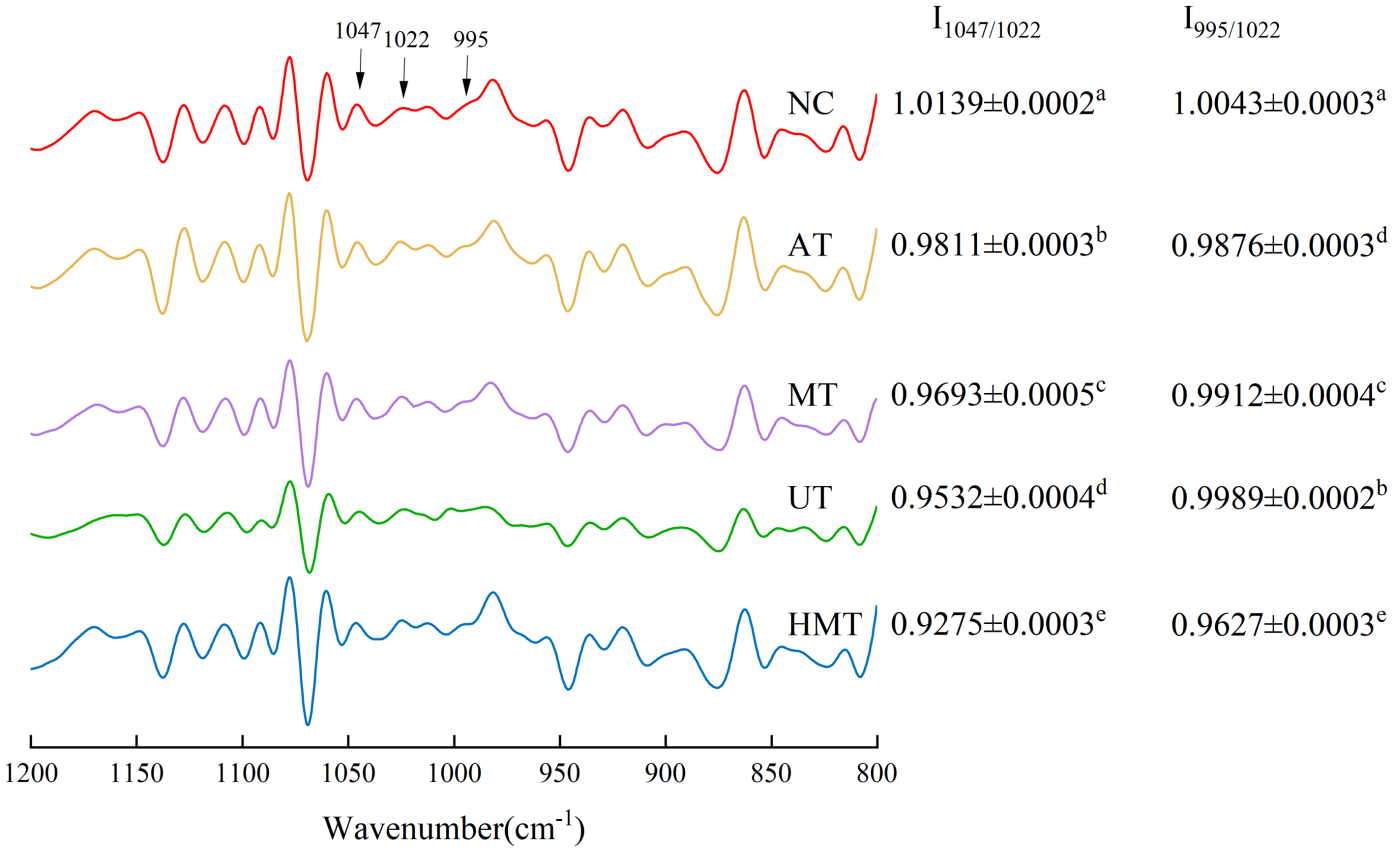
Deconvoluted FTIR spectra of five samples.

The Fourier transform near-infrared spectra and the ratio of I_1047/1022_ and I_995/1022_ for NC, AT, MT, UT, and HMT are shown in Figure 2. The band ratio at 1047 cm^−1^ and 1,022 cm^−1^ quantifies the short-ranged degree of order, and the band ratio at 995 cm^−1^ and 1,022 cm^−1^ can be used to assess the degree of the double-helix (21). The I_1047/1022_ and I_995/1022_ for NC was 1.0139 and 1.0043, which was higher than that I_1047/1022_ of 0.68 in a previous study on native banana flours (46).

The I_1047/1022_ and I_995/1022_ ratios for different treatment methods had significant differences (*p* < 0.05). I_1047/1022_ were observed in the following order: AT (0.9811), MT (0.9693), UT (0.9532), and HMT (0.9275). According to a previous study (43), the FT-IR results indicated that a greater degree of helical organization in the amorphous parts resulted in a higher molecular order in AT sample. These differences may be ascribed to the fact that the AT is an internal treatment that results in a surface order that is not severely disrupted (47). Microwaves vibrate water and starch molecules, resulting in temperature increases that disrupt starch surfaces (9). The cavitation effect of ultrasound effect severely damages starch granules surfaces, reducing I_1047/1022_ (48). The HMT samples had the lowest I_1047/1022_ that might have been caused by heat and stirring, leading to the greatest destruction of the surface order. Furthermore, based on Lin et al. (49) research, the short-range order structure could be ascribed to the pectin and lipids bound with starch *via* non-covalent interactions that form V-type starch complexes. This could explain the higher I_1047/1022_ in AT, owing to the high content of pectin and lipids in AT (Table 1). I_995/1022_ were observed in the following order: UT (0.9989), MT (0.9912), AT (0.9876), and HMT (0.9627). The value of I_995/1022_ in the UT sample was higher than that of MT, AT, and HMT, indicating a more ordered external region structure. UT promote the formation of double-helix to make the double-helix structure more compact and orderly, which was less susceptible to attacked by digestive enzymes, improving the RS content of UT sample compared with other pre-gelatinized samples.

The I_1047/1022_ of the pre-gelatinized samples were lower than NC, indicating that the molecular order of the different treatment samples was weaker than that of NC. According to Zhang et al. (50), the structures of the different treatment samples were severely damaged, which induced a structure reorganization during the retrogradation process. However, the reorganized structures of the different treatment samples had fewer orders than those of the NC synthesized using natural enzymes.

### 3.7. X-ray diffraction

**Figure 3:**
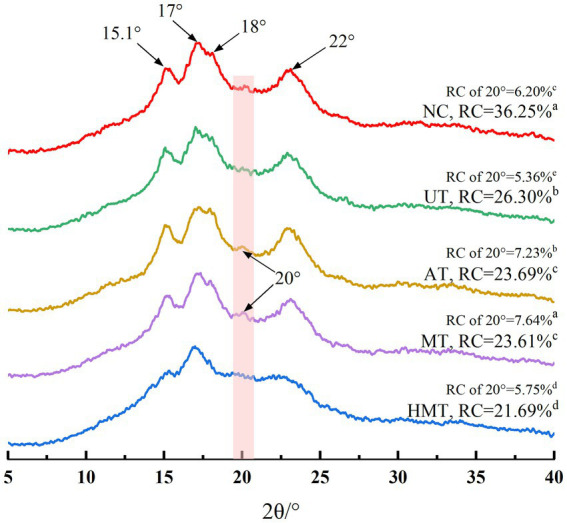
X-ray diffraction patterns of five samples.

XRD was used to further investigate the impact of the treatments on the long-range crystalline structures of the samples. The crystallinities of the native and pre-gelatinized banana flours are shown in Figure 3. The diffraction peaks at 15.1°, 17°, 18°, and 22° (2θ) of NC were recognized as those of C-type crystalline structures, based on Kumar et al. (51) study. The crystalline properties of C-type crystalline structure in banana flours were consistent with those reported in a previous study (24). The RC of NC was 36.25%, which was similar with the previous results (31.9%) (52).

The intensities of the diffraction peaks at 15.1°, 17°, 18°, and 22° in the pre-gelatinized starches, AT, and MT have an additional small peak at 20°. According to Cahyana et al. (53) study, the XRD patterns of UT showed C-type starch characteristics, whereas those of AT and MT were C + V-type, and HMT was A-type. The order of the crystalline domains at approximately 20° was MT (7.64%) > AT (7.23%) > NC (6.20%) > HMT (5.75%) > UT (5.36%). These differences may be due to an increase in shear conditions during autoclave and microwave processing that formed a complex between amyloses and lipids (21, 54). Table 1 shows that the lipid content was 0.42 and 0.40% for MT and AT, respectively. Although the lipid and protein contents in AT were higher than those in MT, the RC around 20° of MT was higher than that of AT, which might be attributed to the greater impact of MT than that of AT. In addition, severe UT and HMT resulted in the release of associative pectin and other ingredients, which prevented the formation of a V-type crystalline structure between lipid and starch molecules, resulting in lower RC at approximately 20° in UT and HMT samples than in MT and AT samples. Noteworthily, the formation of V-type starch complexes mainly through amylose apparently decreases the apparent amylose content values, the glycosidic bond has a higher tendency to break than to form the V-type complex, resulting in an increase in the apparent amylose content.

The RC values were in the following order: UT (26.30%) > AT (23.69%) > MT (23.61%) > HMT (21.69%), and there were significant differences among the pre-gelatinized samples. The UT conditions appeared to favor the formation of a relatively organized crystalline region, increased crystalline perfection, and the generation of new crystallites formed by interactions among starch chains, which may explain why the RC of UT was higher than that of the other samples. This result was consistent with the gelatinization temperatures (Table 3). According to Oyeyinka et al. study (55), the AT removes the amylopectin-based ordered structure and generates an amylose-based ordered structure, which damages the ordered crystalline structure, resulting in an imbalance in the lamellar matrix. As a previously report (56), owing to the disruption of the multilayer arrangement of starch, the relative crystallinity decreased and the double-helix structure of the amylopectin crystallized and the area became unstable after MT. The peak intensity may have decreased owing to the loss of the crystal region, which may have caused the cleavage of hydrogen bonds. The crystalline structure of the HMT was A-type, its RC was the lowest compared to that of the other treatments, and its principal peaks were dispersed. This was because pasting gelatinization completely destroys the starch crystal structure and breaks the molecular chains. This result was consistent with that of the measurement in ΔHg values (Table 3), which could be attributed to HMT severely destroying the structure and forming smaller fragments that could not be used as crystal nucleus, resulting in fewer double-helices and long-range ordered structures during the retrogradation process. The UT had a higher relative crystallinity than other treatment samples so that it had a lower digestion speed than different samples. This result also explained the higher RC in UT led to the higher HI and GI than those in AT, MT, and HMT. In addition, low short-range ordered structures were observed in UT (Figure 2), demonstrating UT damaged the starch granules surfaces and induced formation of a relatively organized crystalline region.

The RC of the pre-gelatinized samples was lower than that of NC, which was possibly due to the disruption of double-helix crystals by the different treatments, which was demonstrated as a decrease in ΔHg in the DSC results (Table 3). Compared with NC, the structures of the four pre-gelatinized samples retrograded significantly, reducing the RC value (Figure 3), which was consistent with the decrease in samples in FTIR. Wang et al. reported (57) that the RC of potato starch decreased from 4.9–32.5%, after a high-pressure treatment. Jiang et al. (58) found that the RC value dropped from 34.24–29.81% upon MT of starch. A similar reduction of 32.39–31.64% was recorded in a previous study (8) on corn starch subjected to UT. The RC of proso millet starch was also reported to decrease from 22.68–20.88% after HMT (59).

### 3.8. Scanning electron microscopy

**Figure 4:**
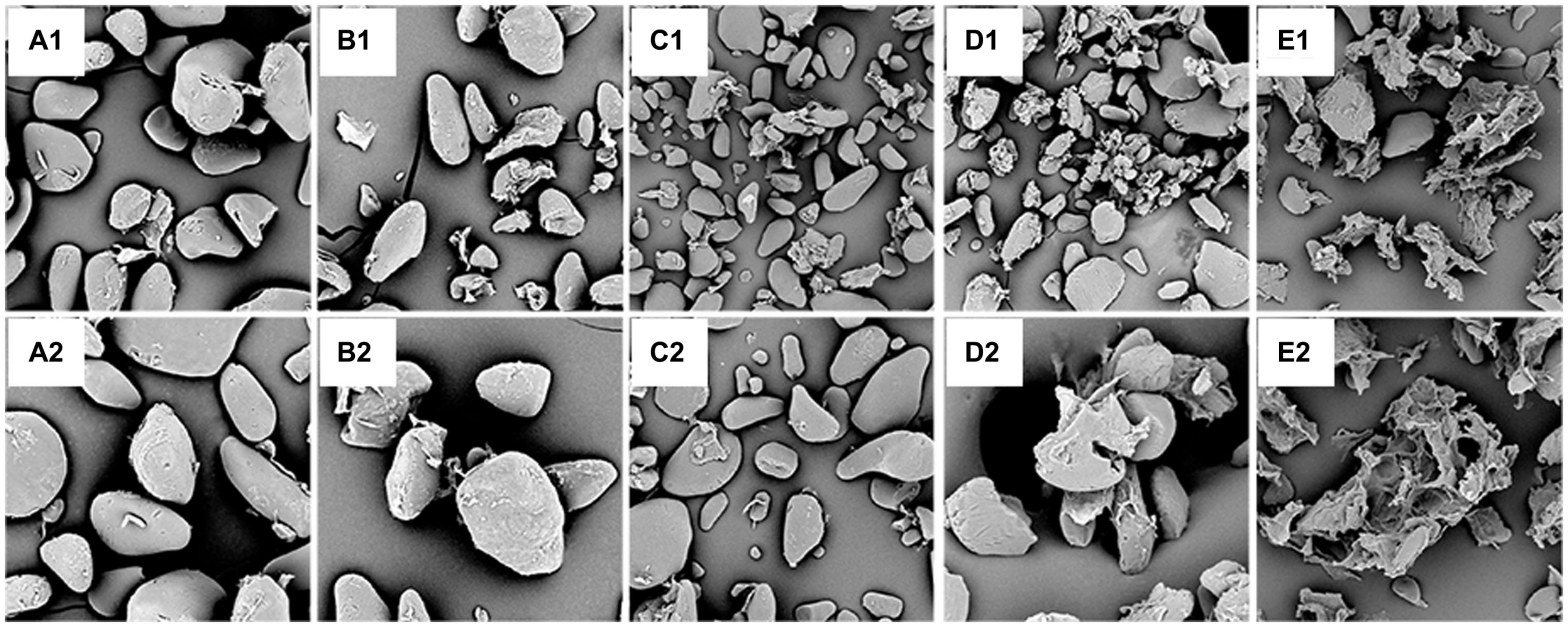
Scanning electron micrograph of different samples: **(A)**: NC, **(B)**: AT, **(C)**: MT; **(D)**: UT; **(E)**: HMT, (1): 1500×, (2): 2500 × .

The morphological structures of the samples from native and pre-gelatinized unripe and inferior banana flours were examined using SEM, as shown in Figure 4. As shown in Figure 4A, the NC granules had irregular, elongated, and spheroidal shapes and sizes, and some pectin was attached to the smooth granule surface. According to Li et al. study (60), these characteristics prevent effective enzymatic hydrolysis, resulting in poor digestion rates.

As shown in Figure 4B, some broken particles and rough surfaces appeared in the AT, which can be ascribed to the effect of steam under pressure during autoclaving. This morphological change in the AT may be due to the aggregation of linear starch fragments and retrogradation occurring during repeated cycles of autoclaving and cooling. In the MT sample (Figure 4C), some fractions had a regular structure and were intact, starch granules appeared concave and wrinkled, and more fragments were observed. A small number of starch granules were broken, and some starch granules were combined. This may be due to the loss of water due to the high internal steam pressure developed by moisture during rapid microwave heating (61). Loss of the physical integrity of the starch was observed in UT (Figure 4D), and many deposits, cracks, and grooves were observed on the surface of the starch granules. A possible reason may be that the cavitation bubble collapses rapidly, producing cracks and grooves on the granule surface during UT. Both UT and HMT had a severe effect on the starch that would be fractured and resulted in peeling off the proteins and pectin from the starch granules, but HMT samples were heavier and formed increasingly smaller fragments so that more crystals could not be formed (62). Compared with AT and MT, UT had a stronger force to separate more free proteins and pectin. Although UT produced more fragments, and the lipid content in UT samples was lower than that in AT and MT samples; the fragments formed complexes with other components and increased the relative crystallinity. After the HMT, the surface granules were severely damaged and showed large amorphous holes (Figure 4E). The pre-gelatinized samples presented rough surfaces, and UT and HMT samples contained large holes. The SEM results could be used to explain the order of I_1047/1022_ value of the pre-gelatinized samples. The rough surfaces of HMT may have contributed to its lower resistance to enzymatic erosion than those of AT, MT, and UT (Table 2). The UT samples ruptured on ultrasonography and released the other components. During the retrograde, the starch and other components would form complexes that were resistant to hydrolysis, according to previously reported results (63).

Compared to the NC, the different treatments severely destroyed the surface granules, resulting in granules of varied large sizes that were practically amorphous, with various forms and emulsion bumps (Figures 4B–E). The surfaces of the samples after different treatments were rough and porous, and UT and HMT lost their original starch particle morphologies. These characteristics will enable the digestive enzymes to reach starch granules more easily than in case of NC. The microscopy results further verified that the surface granules were damaged after the different treatments, leading to a poor digestion rate (Table 2).

## 4. Conclusion

In this study, AT, MT, UT, and HMT were applied in unripe and inferior banana flours, and studied its physicochemical and *in vitro* digestive properties. The total starch, soluble sugar, RS, and apparent amylose content of the pre-gelatinized unripe and inferior banana flours varied significantly among the treatments. The physicochemical and structural properties were strongly related. In addition, AT and MT had a C + V-type crystal structure, UT maintained the C-type, and HMT change to A-type. The pre-gelatinized flours granules contained irregular oval-shaped granules with rough surfaces, and low RC, I_1047/1022_ and I_995/1022_. In four physical treatments, the UT sample had a higher RS content and thermal gelatinization temperatures, a lower degree and rate of hydrolysis, and a more crystalline structure. Therefore, UT may be more suitable for processing unripe and inferior banana flours. The study results demonstrated the possibility of using various treatment methods for potential application of unripe and inferior banana flours in the food industry.

## Data availability statement

The original contributions presented in the study are included in the article/Supplementary material, further inquiries can be directed to the corresponding authors.

## Author contributions

SZ: methodology, investigation, writing-original draft preparation, and data curation. KaZ: conceptualization, software, visualization, and formal analysis. FX: resources and funding acquisition. KeZ and XC: supervision, validation, and visualization. YZ: resources, project administration, funding acquisition, visualization, and writing – review and editing. GX: validation. All authors contributed to the article and approved the submitted version.

## Funding

This research was funded by the Key Research and Development Program of Hainan Province (ZDYF2022SHFZ122), the Chinese Central Public Interest Scientific Institution Basal Research Fund (1630142022007). This study was financially supported by the Spice and Beverage Research Institute, Chinese Academy of Tropical Agricultural Sciences and the Key Laboratory of Processing Suitability, and Quality Control of the Special Tropical Crops of Hainan Province.

## Conflict of interest

The authors declare that the research was conducted in the absence of any commercial or financial relationships that could be construed as a potential conflict of interest.

## Publisher’s note

All claims expressed in this article are solely those of the authors and do not necessarily represent those of their affiliated organizations, or those of the publisher, the editors and the reviewers. Any product that may be evaluated in this article, or claim that may be made by its manufacturer, is not guaranteed or endorsed by the publisher.

## Abbreviations

RS2: type-2 resistant starch
RS: resistant starch
RDS: rapidly digestible starch
DSC: differential scanning calorimetry
XRD: X-ray diffraction
SEM: scanning electron microscopy
NC: native banana flour
AT: autoclaving treatment
MT: microwave treatment
UT: ultrasound treatment
HMT: heat-moisture treatment
SDS: slowly digestible starch
HI: hydrolysis index
GI: glycaemic index
ANOVA: one-way analysis of variance
RC: relative crystallinity
